# Reproducibility of goniometric measurement of the knee in the in-hospital phase following total knee arthroplasty

**DOI:** 10.1186/1471-2474-8-83

**Published:** 2007-08-17

**Authors:** Anton F Lenssen, Ellen M van Dam, Yvonne HF Crijns, Mark Verhey, Ruud JT Geesink, Piet A van den Brandt, Rob A de Bie

**Affiliations:** 1 University Hospital Maastricht, Department of Physical Therapy, Maastricht, The Netherlands; 2 University Hospital Maastricht, Department of Orthopaedics, Maastricht, The Netherlands; 3 Maastricht University, Department of Epidemiology, Maastricht, The Netherlands; 4 Caphri Research Institute, Maastricht, The Netherlands

## Abstract

**Background:**

The objective of the present study was to assess interobserver reproducibility (in terms of reliability and agreement) of active and passive measurements of knee RoM using a long arm goniometer, performed by trained physical therapists in a clinical setting in total knee arthroplasty patients, within the first four days after surgery.

**Methods:**

Test-retest analysis

Setting: University hospital departments of orthopaedics and physical therapy

Participants: Two experienced physical therapists assessed 30 patients, three days after total knee arthroplasty.

Main outcome measure: RoM measurement using a long-arm (50 cm) goniometer

Agreement was calculated as the mean difference between observers ± 95% CI of this mean difference. The intraclass correlation coefficient (ICC) was calculated as a measure of reliability, based on two-way random effects analysis of variance.

**Results:**

The lowest level of agreement was that for measurement of passive flexion with the patient in supine position (mean difference 1.4°; limits of agreement 16.2° to 19° for the difference between the two observers. The highest levels of agreement were found for measurement of passive flexion with the patient in sitting position and for measurement of passive extension (mean difference 2.7°; limits of agreement -6.7 to 12.1 and mean difference 2.2°; limits of agreement -6.2 to 10.6 degrees, respectively). The ability to differentiate between subjects ranged from 0.62 for measurement of passive extension to 0.89 for measurements of active flexion (ICC values).

**Conclusion:**

Interobserver agreement for flexion as well as extension was only fair. When two different observers assess the same patients in the acute phase after total knee arthroplasty using a long arm goniometer, differences in RoM of less than eight degrees cannot be distinguished from measurement error. Reliability was found to be acceptable for comparison on group level, but poor for individual comparisons over time.

## Background

Total knee arthroplasty (TKA) is a common orthopaedic procedure. In the Netherlands, over 18000 TKAs are performed annually [1] After the operation, many patients require physical therapy (PT) to regain functional independence and resume work-related physical activities [2]. One of the main components of the PT programme is mobilisation of the knee joint to increase the range of motion (RoM).

Physical therapists and orthopaedic surgeons use measurement of RoM not only to quantify limitations at the start of treatment but also as an outcome measure to justify their actions or quantify the effectiveness of interventions.

The use of validated and reproducible measurement instruments is an important prerequisite for the evaluation of clinical practice, as well as for the interpretation of study results [3]. The long-arm universal goniometer (UG) is an instrument frequently used to quantify restrictions in range of motion (RoM) [4,5]. Over the years, many studies [3-12] have addressed the reproducibility of RoM measurements of the knee. Studies [3,6] have shown that goniometric measurements are more reliable than visual estimates. Several authors reported intraobserver reproducibility to be better than interobserver reproducibility [6,9]. Generally speaking, reproducibility is better for knee flexion than knee extension [5,10,11].

In our review of the literature most studies have reported on the reliability of RoM measurements, whereas only one study also reported on interobserver agreement [11]. Agreement between observers is an essential component of reproducibility of measurement when utilized in a clinical setting. A method to describe clinically acceptable agreement between observers was introduced by Bland and Altman [13]. The method is based on simple calculations of the standard deviation of the difference between two observers and plotting test differences against their mean [14]. The magnitude of the SD expresses the extent to which the observers are able to achieve the same value [15]. Subsequently, the 95% limits of agreement are calculated, defined as the mean difference between observers ± 1.96 SDs of this mean difference. The limits of agreement can be seen as the range of expected measurement error. Meaningful change in measurement would be reflected by values falling outside this range. Only differences that exceed the limits of agreement will be interpreted as "real" differences exceeding measurement error. The smallest difference outside the 95% range is called the smallest detectable difference. Deciding whether the magnitude of the limits of agreement, representing acceptable agreement, is meaningful is a clinical rather than a statistical decision.

It is important in reproducibility research to take into account what is to be measured, by whom and in what situation, because results of any kind of research will primarily be valid for this particular situation. Too often, the focus is on the measurement tool itself, whereas observers, patient categories and test situations are neglected [16].

Most of the existing reports we found in our literature review on the reproducibility of RoM measurements of the knee relate to studies on native knees. Only Edwards et al [17] reported on the reproducibility of measurements in patients after TKA implantation. Several studies [5,17] have measured in a laboratory-type environment or test protocol [10]. In clinical practice, patients are seen by different therapists and orthopaedic surgeons during their hospital stay. All of them measure range of motion to evaluate progress in the first days after surgery. However, the reproducibility of RoM measurements may vary with the clinical problem, the examiner and the environment in which reproducibility is measured.

In the present study, active and passive RoM was measured to reflect clinical practice. Gajdosik et al [4] state that passive movements are more difficult to reproduce because of the stretching of soft tissues. The limits of range of motion depend on the force applied to the limb. The objective of the present study was to assess interobserver reproducibility (in terms of reliability and agreement) of active and passive measurements of knee RoM using a long arm goniometer, performed by trained physical therapists in a clinical setting in total knee arthroplasty patients, within the first four days after surgery.

## Methods

### Patients

Between January 1 and March 30, 2004, consecutive eligible hospitalised patients in the acute phase after total knee arthroplasty at the orthopaedic ward of the Maastricht university hospital were invited to participate in the study by the first author (AFL). Patients were eligible if they met the following inclusion criteria: status after TKA because of osteoarthritis, ability to co-operate (sufficient Dutch language skills, no dementia) and having given informed consent. Patients with a history of neuromuscular pathologies and patients with revision TKA were excluded.

The study was part of a larger trial approved by the Maastricht University and University Hospital medical ethics committee.

### RoM measurement

Two experienced observers (YC & MV), respectively working 3 and 5 years at the orthopaedic department of a university hospital, both physical therapists, independently measured active and passive flexion and extension RoM of the operated knee using a long-arm goniometer. They were kept unaware of the measurement data of their counterpart. Measurement procedures were standardised prior to the study. Both therapists had trained the procedures on 30 healthy subjects before the start of the trial.

All measurements were taken on the third or fourth day after surgery. Observers used a predefined procedure for the measurements (Table 1).

**Table 1:** Measurement procedure

Knee	Patient position	Bony orientation
Flexion	Seated, hips in 90° of flexion	Proximal trochanter major Lateral femur epicondyle
Flexion	Supine	Caput fibulae
Extension	Supine	Lateral malleolus

To prevent the occurrence of systematic differences between observers because of repeated testing, the test sequence, and thus observer order, was randomized. The time interval between the measurements by the first and second observers was less than five minutes, and the patients did not receive any therapy between the two measurements. Only one observer was present in the examination room at any time, together with a research assistant. The research assistant recorded the number of degrees reported by the observer. Observers and patients were kept unaware of the measurement outcome generated by the previous observer.

RoM was measured using a long-arm goniometer (arm length 50 cm, Enraf-Nonius™). Active RoM was determined prior to passive RoM for each particular motion.

Patients recorded severity of pain while being tested by giving a report mark ranging from 0 'no pain' to 10 'severe pain'.

**Figure 2:**
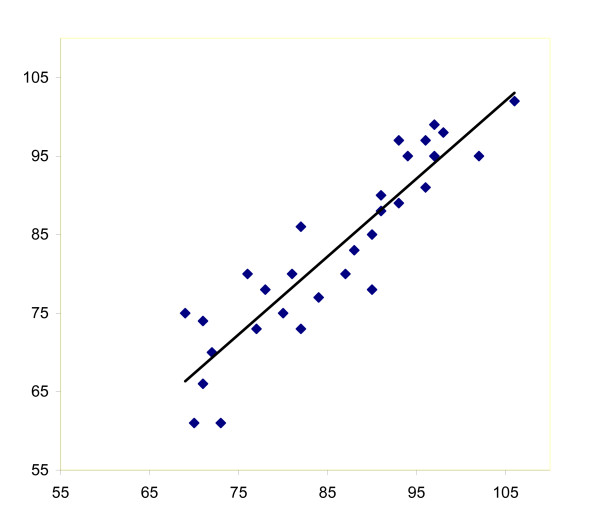
Scatter plot of interobserver reliability of measurement of passive flexion whilst sitting, as indicated by the ICCs.

**Figure 3:**
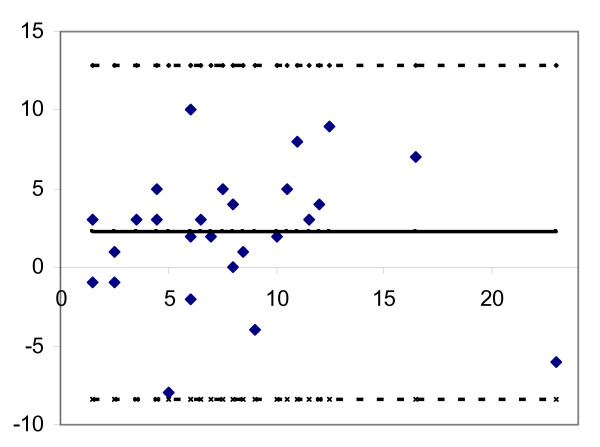
Differences between observers, plotted against the mean values of both observers for each patient for passive extension. The figure shows the mean difference between observers (solid line at centre) and the limits of agreement (dashed outer lines corresponding to ±1.96 SD of the mean difference between the first and second observers).

Measurements were taken while the patients were supine on a hospital bed with both legs resting on the bed. The final step was to identify and mark the bony landmarks on the subjects to standardize the goniometer placement and to facilitate measurements. The bony landmarks identified were the greater trochanter, the lateral femoral condyle, and the lateral malleolus. All the participants were tested in knee flexion and in knee extension (Figure 2). While maintaining a supine position, the subject's affected knee was placed in maximum knee flexion to measure extension (Figure 3), the subject remained in the supine position and was asked to extend his/her knee maximally.

Flexion was measured in supine position and while the patient was seated upright on the examination table with hips in 90° of flexion. Whereas most studies [5,7,17] have measured flexion in supine position, at our hospital we tend to measure flexion while the patient is seated. We therefore measured flexion RoM in both positions.

All goniometric measurements were performed according to the technique described by Norkin and White [18], with the centre of the fulcrum positioned over the lateral condyle of the femur. The proximal fixed arm of the goniometer was aligned with the axis of the femur by using the greater trochanter as a reference point. The distal mobile arm was aligned using the lateral malleolus.

### Statistical analysis

For each observer, the mean and standard deviations were calculated for each RoM assessed. To quantify reproducibility, we distinguished two different types of reproducibility measures with different interpretations: measures of agreement and measures of reliability. Measures of agreement refer to the absolute measurement error (presented in the units of measurement of the instrument) that is associated with one measurement taken from an individual patient [19,20]. Measures of agreement provide insight into the ability of two or more observers to achieve the same value. Measures of reliability refer to the relative measurement error, i.e. the variation between patients in relation to the total variance of the measurements. They provide insight into the ability of two or more observers to differentiate between subjects in a group.

### Agreement

The mean difference between the two observers and the SD of this difference were calculated. The magnitude of the SD expresses the extent to which the observers were able to achieve the same value [15]. Subsequently, the 95% limits of agreement were calculated, defined as the mean difference between observers ± 1.96 SD of this mean difference [13].

### Reliability

The intraclass correlation coefficient (ICC) is defined as the ratio of the variance between patients to the total variance. ICC values can theoretically range from 0 to 1, with a higher value indicating that less variance is due to other factors such as differences between observers. An intraclass correlation coefficient of at least 0.70 is considered to be satisfactory for group comparisons, and a value of 0.90–0.95 for individual comparisons [21]. The ICC was calculated from a two-way random effects model, for absolute agreement.

We used SPSS 12.0 statistical software to calculate the ICCs [22]

## Results

Over a period of three months (January to April 2004) 30 TKA patients was recruited. The mean age of included patients was 69 years (range 51–77) and 80% were female. TKA of the left knee had been performed in 17 cases, while 13 patients had a right knee TKA. All measurements were taken three or four days after surgery.

**Figure 1:**
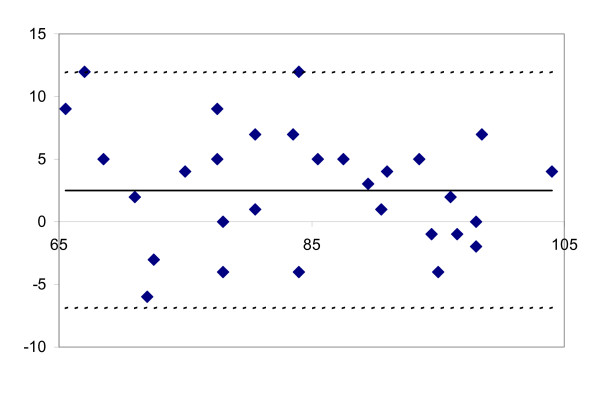
Differences between two observers, plotted against the mean values for both observers for each patient for flexion RoM in sitting position. The figure shows the mean difference between observers (solid line at centre) and the limits of agreement (dashed outer lines corresponding to ±1.96 SDs of the mean difference between the first and second observers).

**Figure 4:**
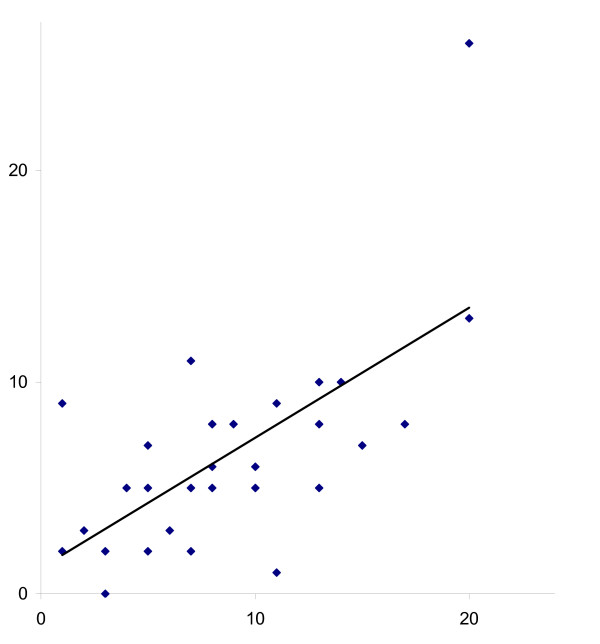
Scatter plot of interobserver reliability of measurement of passive extension.

The results of the interobserver agreement and reliability with regard to the different RoM measurements are presented in table 2 and figures 1, 2, 3, 4. Remarkably, both observers measured higher flexion angles with patients in sitting position.

**Table 2:** Inter-rater reproducibility of knee RoM measurement

Tested movements	Observer A (degrees)		Observer B (degrees)		Agreement observer A-B			ICC (95%CI)
	mean ± sd		mean ± sd		md ± sd		LoA*	
Active Flexion sitting	81.4	10.7	78.3	11.3	3.1	5.3	-7.5 – 13.7	0.86 (0.64–0.94)
Passive flexion sitting	85.8	10.5	83.1	11.4	2.7	4.7	-6.7 – 12.1	0.88 (0.69–0.95)
Active flexion supine	70.6	13.2	70.5	17.0	0.06	7.3	-14.6 – 14.7	0.89 (0.78–0.95)
Passive flexion supine	79	12.8	77.6	17.0	1.4	8.8	-16.2 – 19	0.88 (0.77–0.94)
Active extension	11.1	5.0	10.2	4.7	0.9	4.1	-7.3 – 9.0	0.64 (0.38–0.81)
Passive extension	8.8	5.1	6.6	4.8	2.2	4.2	-6.2 – 10.6	0.62 (0.28–0.80)

Sd, standard deviation; md, mean difference; CI, confidence interval; LoA, limits of agreement, calculated as the mean difference between observers ± 1.96SD of this mean difference.

### Agreement

The observers produced quite similar measurements for extension and flexion in the supine position; however, observer A measured a consistently wider range of motion than observer B.

The highest level of agreement was found for active and passive extension and passive flexion whilst sitting (Figure 1). Limits of agreement ± 1.96 SD were 3.2 ± 9.4 degrees for passive flexion and 0.9 ± 8.2 degrees for active extension.

Lowest levels of agreement were found for active and passive flexion whilst supine. Limits of agreement ± 1.96 SD were 0.06 ± 14.6 degrees for active flexion and 2.2 ± 17.6 degrees for passive flexion.

Figure 1 shows the difference between observers, plotted against the mean value for both observers for passive flexion measured in a sitting position and for passive extension measured in supine position. Figures 1 and 3 both show that errors of the measurement were independent of the magnitude of the range of motion (homoscedasticity).

### Reliability

ICC values were highest for active and passive flexion whilst sitting (Figure 2), and lowest for active and passive extension. The ICC values ranged from 0.62 for passive extension to 0.89 for active flexion. The ICC for extension was lower than the ICCs for flexion, irrespective of active or passive measurement.

Pain during RoM measurement, as assessed by a 11 point scale, was worse in flexion and in passive measurements. No differences between the two observers were found either for perceived pain or for perceived pain and RoM reached during measurement (data not presented).

### Smallest detectable difference

Based on the results of the interobserver agreement, the smallest detectable differences would lie between 8.2 degrees for active extension and 17.6 degrees for passive flexion while supine. This means that only changes in knee range of motion larger than these values can be detected beyond measurement error when different clinicians perform RoM measurements in a comparable clinical environment.

## Discussion

This study investigated the interobserver reproducibility of the assessment of active and passive RoM of the knee in patients after TKA. The results show that there was considerable variation in the agreement between observers in all movements tested. The lowest limit of agreement was found for active extension. As regards flexion, the lowest limit was found for the measurement of passive flexion whilst sitting with 90° flexion of the hip.

With respect to individual measurement over time ICC values have to be considered as poor.

However for group comparison the reliability of the flexion measurement was satisfactory for group comparison with exception of the measurement of extension. This may have been caused by the wider RoM when measuring flexion. The ICC depends on the range of the true quantity in a sample, so if this range is wide, the correlation will be greater than if it is narrow [23].

A similar explanation can be given for the narrower limits of agreement found for extension. The range of outcomes for extension is generally narrow, leading to narrower limits of agreement.

The results show that both observers measured less flexion RoM with patients in supine position than in sitting position. A possible explanation for the difference in measurement positions may be that the supine position is less stable and allows more degrees of freedom of movement in both hip and knee compared to the sitting position, in which the upper leg is fixed on the examination table, as already indicated by Gajdosik [4].

Linden-Peters et al. [11] were the first to report on agreement in the measurement of knee RoM.

They reported better results for extension measurements. We believe this is probably caused by differences in the selection of the study population. Our population had a wider range of extension, leading to a wider limit of agreement.

Although the measurement procedure was standardised, differences in effort by the patient are a potential source of bias in these measurements, and the influence of the examiner is added as a possible source of variation when measuring passive RoM. The amount of force used by the different examiners to reach full RoM may well influence the reproducibility of the measurements [4].

Unlike those of others [5,10,11] our results do not show any differences between active and passive measurements. This might be caused by the level of experience, and the training of the participating therapists prior to the study, as well as the standardisation of measurements used by all therapists working with TKA patients in our hospital. Another explanation may be that in the acute phase after surgery, patients tend to guide passive measurement, for fear of extreme pain when going into extreme flexion or extension. This may well cause measurements of passive and active RoM to be more alike.

Although we concentrated on measuring in a clinical situation, our results with respect to reliability are similar to results reported in the literature, which were obtained in a laboratory environment [5,6,10,12,17]. This may have been partly caused by the use of a predefined testing procedure by experienced therapists, who had trained the procedures on healthy subjects and patients before the start of the study. Van Genderen et al. [16] also described positive effects of including these steps in the measurement design.

Hence, we believe that RoM measurement in a clinical situation is possible without loss of reliability. However, it may be questioned whether agreement is not of greater importance in clinical measurement situations.

### Limitations

The decision to use only two testers in this study might be debatable. For a correct simulation of everyday practice, we would have preferred to include all staff members involved in the follow-up of TKA patients at our clinic. We chose to include only two testers, however, because we believe that the inclusion of agreement is of the utmost importance in analyses of measurement in a clinical situation. This inclusion of limits of agreement as an outcome was only possible when using two testers.

Our research focused on the difference between observers, and did not include intraobserver reproducibility. We believe that studying intraobserver reproducibility would involve more interference with everyday practice, as individual observers would need to perform multiple measurements on the same patient and would have to be blinded for the outcome of each of their measurements. This would interfere with our intention to mimic clinical measurement procedures and not to create a laboratory environment for our measurements.

In spite of our use of a measurement procedure with standardised measurement and pre-study training, we still found differences in RoM measurement between the two observers. Observer A persistently measured greater flexion RoM. We believe that, despite training of the observers this might still be caused by persistent differences between the testers in the choice of the fulcrum of rotation. Brosseau et al. [5,9]already mentioned the choice of the fulcrum as being the Achilles heal of RoM measurement in the knee. This problem might be overcome by using the parallelogram goniometer introduced by Brosseau et al [5,9].

We didn't standardise the amount of force used for measurement of passive RoM. This may be a cause for the differences we found between observers.

Since this study was conducted in one physical therapy department, by therapists who measure RoM in TKA patients daily, the results may not necessarily be generalizable to all physical therapists.

### Relation between reproducibility and responsiveness

To be useful for outcome assessment in clinical practice or research, an instrument should have high responsiveness, which is strongly related to the level of agreement [24] Limits of agreement should be smaller than the minimum clinically relevant difference one wants to detect. As regards clinical practice, the large limits of agreement for all measurements in our study indicate that we should be very careful in comparing and interpreting results obtained by different examiners. As regards research, we suggest to try and use only one observer. Unfortunately, practical reasons make it very difficult to investigate the level of intraobserver reproducibility. Future studies should investigate whether further standardisation or the use of other measurement tools such as a parallelogram goniometer might lead to smaller limits of agreement.

## Conclusion

Interobserver agreement was low in the assessment of active and passive RoM of the knee in patients in the acute phase after total knee arthroplasty, notwithstanding the fact that it was measured by experienced physical therapists. Reliability of RoM measurement was acceptable with regard to group comparisons but poor with regard to individual measurement over time.

## Competing interests

The author(s) declare that they have no competing interests.

## Authors' contributions

AFL and EMvD designed the study, RJTG, PavdB and RAdB participated in the design of the study. AFL, EMvD, YHFC and MV collected the data. AFL performed the statistical analyses and prepared the drafts of the manuscript. All authors read and approved the final manuscript.

## Pre-publication history

The pre-publication history for this paper can be accessed here:



## References

[B1] Biomet, Nederland (2007). http://www.knie.nl/content/knie/knie_kunst.asp.

[B2] Naylor J, Harmer A, Fransen M, Crosbie J, Innes L (2006). Status of physiotherapy rehabilitation after total knee replacement in Australia. Physiother Res Int.

[B3] Rothstein JM, Campbell SK, Echternach JL, Jette AM, Knecht HG, Rose SJ (1991). Standards for tests and measurements in physical therapy practice.. Physical therapy.

[B4] Gajdosik RL, Bohannon RW (1987). Clinical measurement of range of motion. Review of goniometry emphasizing reliability and validity. Phys Ther.

[B5] Brosseau L, Balmer S, Tousignant M, O'Sullivan JP, Goudreault C, Goudreault M, Gringras S (2001). Intra- and intertester reliability and criterion validity of the parallelogram and universal goniometers for measuring maximum active knee flexion and extension of patients with knee restrictions. Arch Phys Med Rehabil.

[B6] Watkins MA, Riddle DL, Lamb RL, Personius WJ (1991). Reliability of goniometric measurements and visual estimates of knee range of motion obtained in a clinical setting. Physical Therapy.

[B7] Kafer W, Fraitzl CR, Kinkel S, Clessienne CB, Puhl W, Kessler S (2005). [Outcome assessment in total knee arthroplasty: is the clinical measurement of range of motion a reliable measurable outcome variable?]. Z Orthop Ihre Grenzgeb.

[B8] Rheault W, Miller M, Nothnagel P, Straessle J, Urban D (1988). Intertester reliability and concurrent validity of fluid-based and universal goniometers for active knee flexion. Phys Ther.

[B9] Brosseau L, Tousignant M, Budd J, Chartier N, Duciaume L, Plamondon S, O'Sullivan JP, O'Donoghue S, Balmer S (1997). Intratester and intertester reliability and criterion validity of the parallelogram and universal goniometers for active knee flexion in healthy subjects. Physiother Res Int.

[B10] Rothstein JM, Miller PJ, Roettger RF (1983). Goniometric reliability in a clinical setting. Elbow and knee measurements. Phys Ther.

[B11] Linden-Peters van den CJ, Genderen FR, Aufdemkampe G (2003). Meten in beweging: betrouwbaarheidsonderzoek van active range of motion (AROM) volgens een andere visie. Nederlands Tijdschrift voor Fysiotherapie.

[B12] Gogia PP, Braatz JH, Rose SJ, Norton BJ (1987). Reliability and validity of goniometric measurements at the knee. Phys Ther.

[B13] Bland JM, Altman DG (1986). Statistical methods for assessing agreement between two methods of clinical measurement. Lancet.

[B14] Tammemagi MC, Frank JW, Leblanc M, Artsob H, Streiner DL (1995). Methodological issues in assessing reproducibility--a comparative study of various indices of reproducibility applied to repeat ELISA serologic tests for Lyme disease. J Clin Epidemiol.

[B15] Terwee CB, de Winter AF, Scholten RJ, Jans MP, Deville W, van Schaardenburg D, Bouter LM (2005). Interobserver reproducibility of the visual estimation of range of motion of the shoulder. Arch Phys Med Rehabil.

[B16] Genderen FR, van Meeteren NLU, Verhoef J (2001). Betrouwbaarheidsonderzoek : van een impliciete receptuur op weg naar een meer expliciete visie ; een overzicht van betrouwbaarheidsstudies van de hand-held dynamometer als voorbeeld. Ned Tijdschr Fysiotherapie.

[B17] Edwards JZ, Greene KA, Davis RS, Kovacik MW, Noe DA, Askew MJ (2004). Measuring flexion in knee arthroplasty patients. J Arthroplasty.

[B18] Norkin CC, White DJ (1995). Measurement of joint motion: a guide to goniometry.

[B19] de Winter AF, Heemskerk MA, Terwee CB, Jans MP, Deville W, van Schaardenburg DJ, Scholten RJ, Bouter LM (2004). Inter-observer reproducibility of measurements of range of motion in patients with shoulder pain using a digital inclinometer. BMC Musculoskelet Disord.

[B20] de Vet HC, Armitage P, Colton T (1998). Observer reliability and agreement. Encyclopedia of biostatistics.

[B21] Scientific Advisory Committee of the Medical Outcome Trust (2002). Assessing health status and quality-of-life instruments: attributes and review criteria. Qual Life Res.

[B22] Norusis (2003). SPSS.

[B23] Bland JM, Altman DG (1995). Comparing two methods of clinical measurement: a personal history. Int J Epidemiol.

[B24] de Vet HC, Bouter LM, Bezemer PD, Beurskens AJ (2001). Reproducibility and responsiveness of evaluative outcome measures. Theoretical considerations illustrated by an empirical example. Int J Technol Assess Health Care.

